# Social health assistance schemes: the case of Medical Financial Assistance for the rural poor in four counties of China

**DOI:** 10.1186/1475-9276-10-44

**Published:** 2011-10-20

**Authors:** Xiao Ma, Juying Zhang, Bruno Meessen, Kristof Decoster, Xiaohui Tang, Yang Yang, Xiaohui Ren

**Affiliations:** 1 West China School of Public Health, Sichuan University, No.17 Section 3 South Renmin Road, 610041. Chengdu, Sichuan, China; 2 Department of Public Health, Institute of Tropical Medicine, Nationalestraat 155, 2000 Antwerp, Belgium

**Keywords:** Health assistance scheme, Medical Financial Assistance, poverty, rural China

## Abstract

**Background:**

Economic transition which took place in China over the last three decades, has led to a rapid marketization of the health care sector. Today inequity in health and poverty resulting from major illness has become a serious problem in rural areas of China. Medical Financial Assistance (MFA) is a health assistance scheme that helps rural poor people cope with major illness and alleviate their financial burden from major illness, which will definitely play a significant role in the process of rebuilding Chinese new rural health system. It mainly provides assistance to cover medical expenditure for inpatient services or the treatment of major illnesses, with joint funding from the central and local government. The purpose of this paper is to review the design, funding, implementation and to explore the preliminary effects of four counties' MFA in Hubei and Sichuan province of China.

**Methods:**

We used an analytical framework built around the main objective of any social assistance scheme. The framework contains six 'targeting' procedural 'steps' which may explain why a specific group does not receive the assistance it ought to receive. More specifically, we explored to what extent the targeting, a key component of social assistance programs, is successful, based on the qualitative and quantitative data collected from four representative counties in central and western China.

**Results:**

In the study sites, the budget of MFA ranged from 0.8 million Yuan to 1.646 million Yuan in each county and the budget per eligible person ranged from 32.67 Yuan to 149.09 Yuan. The preliminary effects of MFA were quite modest because of the scarcity of funds dedicated to the scheme. The coverage rate of MFA ranged from 17.8% to 24.1% among the four counties. MFA in the four counties used several ways to ration a restricted budget and provided only limited assistance. Substantial problems remained in terms of eligibility and identification of the beneficiaries, utilization and management of funds.

**Conclusions:**

MFA needs to be improved further although it evidences the concern of the government for the poor rural people with major illness. Some ideas on how to improve MFA are put forward for future policy making.

## Introduction

For a long time, the international research community has paid relatively little attention to the specific needs of the poorest in low-and middle income countries. Recently however, there has been growing recognition of the miserable life of the chronic poor and of the need to better understand the causes of their exclusion from basic social rights [1]. This increased the interest at country and global level in policies able to assist the poorest in their livelihood. Safety nets and targeted programs have thus been, as policies, gradually rehabilitated in low and middle-income countries [2,3]. The poorest require specific solutions. An entitlement to publicly funded packages is necessary, obviously, but also a specific form of active (and ideally personalized) support, i.e. 'social assistance'.

The health sector is no exception to this rule. In a majority of countries nowadays, households have to contribute with out-of-pocket payments to access health care services [4]. This financial contribution can be substantial and lead households to reduce their health service utilization (or to fall into poverty). The situation of the poorest is particularly problematic, as they cannot afford these out-of-pocket payments. User fee exemption systems do not work in most cases [5,6] and social health insurance schemes tend to exclude the poorest [7], although in some low-income countries, user fee exemption systems have been successful in reducing financial barriers to health care, at least to some extent [8]. Countries are innovating and a growing number of them are developing social assistance schemes to cover the cost of health care for the poorest, i.e. 'social health assistance' [9]. We believe that these initiatives can be important inclusive actions, at a time that the world community declares to be willing to move towards universal coverage.

Recent Chinese developments are in line with the above mentioned trend. In the last three decades, while the Chinese economic success story has caught deservedly the attention of the whole world, health inequity has become an increasingly serious challenge for China. Both the media and academic researchers have frequently reported the inequity issue of health and health care between rural and urban areas, regions, and different economic population groups. Within either urban or rural area, poorer health statues can be observed for low-income population groups. A study on 73, 497 individuals conducted in Hunan province reported that the low-income population suffered more health problems using the indicator of disease concentration index [10]. Differences in health care utilization between population income groups were also found in some studies. In the national health services survey, it was found that the proportion of population who did not use inpatient care when needed was 20% higher for the lowest-income groups than the highest-income groups [11]. It was frequently reported that that poor families often borrow money, sell their productive assets, cut short their children's education in order to pay their medical expenses, or simply do not go to see the doctor when falling ill due to their inability to pay [12]. Within the same region, poor families had heavier financial burden due to disease expenditures than the rich families. In the rural area of western provinces, 7.4% of household incomes were spent on diseases for lowest-income families, while 3.6% of the household incomes went to treatment of diseases for highest-income families [13].

The Chinese government has started to implement a number of policy interventions related to the promotion of equity. Among those policies, the establishment of the New Cooperative Medical System (NCMS) and Medical Financial Assistance (MFA), are essential for improving equity in health in rural China.

The "Decision to further strengthen rural health work" [14] established the NCMS, a government-run voluntary insurance program. Its purpose is to solve the poverty trap due to major illness in the countryside. MFA, the focus of this paper, is another important program that will form an integral component of the new rural health system, and centers on very poor rural households. MFA is expected to alleviate the financial burden from major illness for the poor. It mainly provides assistance to cover medical expenditure for inpatient services or the treatment of major illnesses, with joint funding from the central and local government. By the end of 2006, most counties and cities with rural population had established the scheme [12].

The purpose of this paper is to use a sequential framework developed by Meessen and Criel to explore the effectiveness of MFA in reaching the target group at different stages of program initiation and implementation [15]. Meessen and Criel already applied this framework in the Cambodian context on Health Equity Funding. It assesses whether potential beneficiaries are being 'lost' in several consecutive steps of the targeting process. If necessary, the framework will be adjusted slightly to suit the Chinese MFA context. We hope to give the governments at various administrative levels some advice on how to develop and implement MFA in a more efficient way.

In the first section, the study sites and methods of this study are presented. Next, the analytical framework is introduced. In the results section, we use the analytical framework to shed light on the way MFA targeting unfolds in several consecutive steps. Similarities and differences in design, implementation and management of MFA in the four counties are pointed out. After a short section on the preliminary impact of MFA in the four counties, we conclude with a discussion of the main findings and ways forward.

### Study sites and methods

This study is part of POVILL, a comparative European Union funded research project on the relation between poverty and illness in three Asian countries. In China, the survey rests on four counties: Xiaochang, Hongan, Langzhong and Fushun. The former two are located in Hubei province of central China, the latter in Sichuan province of southwest China. Xiaochang, Hongan and Langzhong are designated as poor counties by the government. This is not the case for Fushun. Table 1 gives some background information about the four counties in 2006.

**Table 1:** Background information of the four counties in 2006

	Xiaochang	Hongan	Fushun	Langzhong
Population (thousand)	643	654	1016	860
Rural population (thousand)	557	544	825	598
Annual income per capita in rural area (Yuan)^a^	2, 051	2, 328	3, 400	2, 889
Threshold for defining a poor family (Yuan/per person per year)	683	720	625	668
Number of medical facilities	387	210	1021	593

^a^1 Yuan = 0.1251 USD on June 30th, 2006; 1 Yuan = 0.0987 EUR on June 30th, 2006

Quantitative data were drawn from the so called Rapid Household Survey (RHS) and official documents of the four counties. In the RHS, 12, 131 households were selected through multistage cluster sampling. First, three townships were chosen randomly from each county; then, 10 villages were selected randomly from each township; finally, about 100 households were selected randomly from each village. RHS included modules on household assets, demographics, health status of household members, utilization of health services, medical expenditures, reimbursement of NCMS and MFA assistance.

Qualitative data were collected through semi-structured interviews conducted by researchers with officials of county health bureaus, county civil affairs bureaus, county hospitals, township health centers and civil affairs offices. In Xiaochang and Hongan, qualitative interviews were conducted in July 2007; in Fushun and Langzhong, interviews were conducted in April 2007. Data and information concerned 2006 though. In each bureau or hospital, we interviewed about two or three key informants. In total, we interviewed about 10 to 15 informants in each county. The interviews focused on MFA policy and aspects, such as the design, implementation, performance and problems. The key information on MFA was collected from county civil affairs bureaus and township civil affairs offices that are in charge of management and implementation of MFA. Supplemental information was gathered from county health bureaus, county hospitals and township health centers, which may be related to MFA indirectly.

### Analytical framework

Targeting is the policy option of concentrating the benefits of an intervention on a pre-identified specific group. Social assistance programs obviously target the poorest. To our knowledge, there is today no clear academic guidance on how to analyze social assistance programs. A large body of literature focuses on the outcomes of the targeting process, but most of these studies are rather silent on implementation issues. In a review of international experiences, Coady, Grosh and Hoddinott noted that the quality of implementation matters tremendously to the targeting outcome and that there was thus an obvious need for further work on implementation. "Program managers need to be able to know more about the details of what was done elsewhere, why the choices were made, how they worked out, and what circumstances affected the outcomes" [3].

Meessen and Criel have put forward an analytical framework built around the main objective of a social health assistance intervention: to have a significant impact for a well identified group of beneficiaries. The framework analyses the intervention in terms of possibilities for the program to have limited impact. The program could fail to reach the target group or bring insufficient assistance to the beneficiaries. They identify six 'targeting' procedural 'steps' which may explain why a specific group does not receive the assistance it ought to receive: (1) the formulation of the intervention; (2) the definition (in measurable terms) of the intended group of benefit; (3) informing the stakeholders, including the target group; (4) the identification of individuals meeting the eligibility criteria; (5) the entitlement; (6) the utilization of the service subsided by the program".

The first step is the programme formulation gathering all the activities related to the initiation of the intervention. Policy makers should clearly formulate their ambition. The target group should be specified and the aim of the intervention should be clarified. In addition, the targeting method and benefit package should be at least broadly defined according to the objectives. Step 2 is the definition of eligibility. In this step, a variety of experts often shows the way forward. It is important to acknowledge that the setting of the eligibility criteria is closely related to the budget available for the intervention. In step 3, the stakeholders are informed: this refers to the different actions taken to inform the different stakeholders about what the programme has to offer. The information about the programme should not only reach actors directly involved, but also all the actors regularly dealing with the target beneficiaries through other programs. Step 4 is the identification of the individuals eligible for assistance. This step amounts to screening a population to identify individuals meeting the eligibility criteria set in the second step. Step 5 is the entitlement, i.e. the action of granting the entitlement for assistance to the sub-population identified in the fourth step. The key issue here is to establish the new status of the individual as a right they can vindicate. Ideally, the entitlement must quickly follow the identification. The entitlement must be clear on the benefit package it gives access to. The last step is the delivery of assistance to the subpopulation entitled in Step 5. The key issue here is to provide assistance that will bring a real benefit for the assisted person. In each of these steps, something can go wrong for potential beneficiaries.

## Results

### Step 1. The formulation of MFA

Starting in October 2002, a series of policies were promulgated by key ministries of the central government, like the Ministries of Health (MoH), Civil Affairs (MoCA) and Finance (MoF), sometimes jointly. Policy measures announced were respectively 'the decision to further strengthen rural health work' [14], 'Proposals on the Implementation of Rural MFA' [16], 'The notice from MoF and MoCA to print and distribute Temporary Measures of Rural Medical Financial Assistance Fund Management' [17], and 'The notice from MoCA, MoH and MoF to promote rural MFA' [18]. Through these series of policies, the rural MFA system was established. With the scheme, the government intends to help rural poor people cope with major illness and alleviate their burden from major illness. Assistance mainly covers medical expenses for inpatient services or the treatment of major illnesses. The program is financed jointly by the central and local governments. Funds from the central government have been used to subsidize provinces in the middle and western regions. Local governments at different levels are required to finance the program according to their economic situation.

MFA is a highly decentralized program, with local governments having discretion over both policy design and implementation. Variations across localities can occur, for example to take into account local circumstances. After the promulgation of the series of decrees on MFA by the central government, the four counties in our study swiftly launched the program. Fushun launched MFA in 2004, and the other three counties started in 2005. The four counties had set up their respective MFA scheme in line with the central policy decrees, adjusting it where necessary to the local situation. The workload was assigned to different bureaus and sections.

The county civil affairs bureau is in charge of the daily operation of MFA, which includes tasks like formulating specific regulation, checking the application of beneficiaries, distributing funds and other management issues of MFA. The township civil affairs offices, which are under the supervision of the county civil affairs bureaus, deal with publicity, identification of beneficiaries and households to be visited; the village civil affairs cadre or village cadre is expected to advertise the scheme, assess the eligibility status of households and identify the beneficiaries.

The county financial bureau is in charge of the collection and supervision of funds. MFA funds are allocated by upper level financial departments to lower level financial departments. Funds are for instance transferred from the central to the provincial level. Regardless of the source, funding is under the supervision of the county financial bureaus through a special bank account.

The county health bureau is responsible for the provision of medical services in a network of hospitals in the four counties. The county health bureau supervises all the hospitals.

No single special department is in charge of supervising these three bureaus (county civil affairs bureau, county financial bureau and county health bureau), but all the departments are supervised by the related department from a higher level. Moreover, the work of the county civil affairs bureau is regularly checked by the county financial bureau. Also, meetings gathering officials from the three departments are organized on a regular basis to discuss MFA related affairs.

The providers of medical services are more rigorously defined in Xiaochang and Hongan, while in Fushun and Langzhong every hospital is acceptable for MFA. In Xiaochang, no coordination with NCMS existed at the time of research because NCMS had not yet been implemented in this county in 2006. In the other three counties, the county civil affairs bureau pays the NCMS premium for the households eligible for MFA, and the county civil affairs bureau and health bureaus are responsible for the coordination between MFA and NCMS.

### Step 2. Defining eligibility

MFA has to combine at least two sets of eligibility, according to economic criteria ('who is poor') and medical criteria (what is a major illness) respectively.

As for the economic criteria, the central government has not provided a specific definition of the 'poor for MFA' so far. In the implementation of MFA, the local governments of the four counties capitalized on other social assistance schemes' regulations to define the 'poor for MFA'. In the sites under study, the main categories of households that are eligible for MFA are those households who were registered in Wubao(Five Guarantee Program), Dibao(Minimum Income Guarantee Scheme), Tekun(Assistance for the Extremely Poor Households) and Youfu(Regulations on Special Care and Treatment for Servicemen), see table 2.

**Table 2:** The criteria for assistance in the four counties in 2006

Criteria	Xiaochang	Hongan	Fushun	Langzhong
Household characteristics	Wubao, Dibao, Tekun, Youfu, the households with special difficulties identified by the county government	Wubao, Dibao, Tekun, Youfu	Wubao, Tekun, the households that became poor due to major illness	Wubao, Dibao, Tekun, Youfu
Health status	Suffered from major illness 1 month before applying Major illness authorized by county government.	Suffered from major illness 3 months before applying Major illness authorized by county government.	Suffered from major illness (defined as an illness with a major impact upon daily life)	Suffered from major illness (defined as: inpatient expenditure of over 5000 Yuan)
Health care expenditure	The out of pocket expenditure is more than 1000 Yuan		None	The inpatient expenditure is more than 5000 Yuan
Other	None		Individuals who get reimbursement from NCMS have priority for MFA	

In Xiaochang, besides these four categories, other households with a family member suffering from major illness or generally leading a hard life can also be included in MFA if their situation is verified by the county government. In Fushun, the criteria are slightly different. Besides Wubao and Tekun, people who sank into poverty due to major illness can also be included in MFA.

There is no national definition of 'major illness' in China at present - and rightly so, given the large variation existing within the country. In Xiaochang and Hongan, the illness categories included in MFA are circumscribed. In Fushun county, major illness is identified as 'illness with a major impact on daily life', such as cancer, nephropathy etc.. In Langzhong county, major illness refers to illness with inpatient expenditure over 5000 Yuan (Table 2). We must note however that in the four counties, major illness is understood as a health problem managed at hospital level.

At first sight, criteria of the MFA seem rather straightforward. However, as discussed under step 4, due to budget constraints, there can be discrepancies between the theory and the practice.

### Step 3. Informing the stakeholders

Once the county civil affairs bureau, county health bureau and county finance bureau have agreed on the regulation documents, the latter are sent to township government or lower level departments directly involved in the implementation of MFA. Besides, copies of regulation documents are also sent to indirect stakeholders such as the Office of the County People's Congress, the Office of the County People's Political Consultative Committee etc.. Although informing the relevant departments is very important, obviously the key stakeholders to contact are poor households (potential beneficiaries). The MFA policy is publicized by the county civil affairs bureaus mainly through websites, banners, scrolls, leaflets, bulletins, New Year pictures, mobile loudspeakers, broadcasting etc. The methods of publicity may vary a little among the four counties according to their local situation. The township civil affairs offices are in charge of publicizing and informing the poor rural households of the MFA scheme. Then the targeted households may apply to the county civil affairs bureaus for assistance or else they can be directed by their neighbor or village officer to the township civil affairs offices. According to official documents, MFA was heavily advertised to poor households in all counties. But, RHS findings show that the awareness rate of MFA was nevertheless rather low in the four counties (Table 3). Only about 11.2% of the poor households were aware of MFA at the time. The reason for this inconsistency between advertising efforts and awareness will be explored in the discussion section.

**Table 3:** Awareness rate of MFA in the four counties in 2006 (RHS)

County	Poor households	Awareness of MFA	
	
	**N(households)**^**a**^	N(households)	Percentage(%)
Xiaochang	202	25	12.4
Hongan	147	16	10.9
Fushun	116	13	11.2
Langzhong	53	4	7.5
	
Total	518	58	11.2

^a ^Including Wubao, Dibao, Tekun, Youfu households and self-report poor households

### Step 4. The identification of individuals meeting the eligibility criteria

This step involves screening an actual population to identify individuals meeting the eligibility criteria set in Step 2. Under step 2, we have reported restrictive but rather clear criteria. However, meeting these criteria may not be sufficient: more often than not, quotas are applied at township level.

In counties where NCMS is in place, the economic criteria are applied ex ante: they are used to identify households of which the NCMS premium will be paid by MFA. Medical criteria are used ex post: they are assessed once a household claims reimbursement for a specific episode of illness. In two of the three counties with NCMS, priority is also given to households who have already received some reimbursement from the NCMS.

When the poor who meet the abovementioned economic criteria receive inpatient treatment or outpatient treatment for major illness as defined by the local government, they are allowed to submit a formal application to the village civil affairs cadre or village cadre for MFA assistance. After getting the application for MFA, the village civil affairs cadre or village cadre makes a household assessment of poverty and diseases by paying a visit to the applicants' homes and their neighbors, checking their certificate of household characteristics, receipt of hospitalization, certificate of discharge from the hospital, NCMS reimbursement certificate. Village cadre discussion takes place as well. Next, the applicants are ranked at township level according to the extent of poverty and medical expenditure. In Fushun and Langzhong, households who receive NCMS reimbursement get priority for MFA assistance over other households. According to the quota of assistance as allocated by the county civil affairs bureau, beneficiaries are identified. After verification by the civil affairs office, certificates are awarded as evidence of MFA qualification. The number of assisted beneficiaries depends on the budget. The approval frequency of assistance is different for the 4 counties: once a month in Xiaochang, once every 2 months in Hongan, no more than 1 month after the application in Fushun, and in Langzhong once during the first half year and once every three months during the last half year.

In MFA regulation, county civil affairs bureaus bear the main financial risk; it comes as no surprise then that their strategy is to only allocate the funds after having received the money from the respective administrative levels. Consequently the identification process tends to be relatively conservative, especially in the first half year, to prevent an MFA fiscal deficit.

In a nutshell, step 4 of the MFA scheme includes both verification (by checking whether the household meets the eligibility criteria) and rationing of the available budget (by ranking households eligible for assistance).

### Step 5. The entitlement

An entitlement can be assessed on two dimensions: the assistance it gives access to and its certainty. As for MFA, we have seen that in three counties, MFA finances the NCMS premium of eligible households. More specifically, those people who meet the economic criterion are entitled to free enrolment to NCMS and will benefit from basic benefit package of NCMS when they are sick and seeking care. This is a secured entitlement. Yet, the benefit package is rather shallow, as the NCMS schemes are practicing rules (e.g. deductibles, cap on reimbursement.) that severely restrict the proportion of health care expenditure those households get reimbursed. Those people meeting both economic criteria and medical criteria will benefit from additional support (direct cash assist after treatment) by MFA.

As shown in table 2 MFA is a scheme entitling households to assistance for their utilization of hospital services, or even some designated hospitals. In terms of security of this entitlement, the reimbursement by the MFA scheme is not guaranteed. First there is an obligation to claim rapidly one's reimbursement (Table 2). In Xiaochang County, the major illness must have occurred one month before the application. Second, because of the rationing (through quotas), an eligible household may not get the assistance which it is entitled to.

Practically speaking, the entitlement takes the format of a certificate issued by the county civil affairs bureau. As already mentioned, it can take some time to receive this certificate. The applicants can get cash assistance through the MFA certificate from the civil affairs bureau, the township civil affairs office or a bank identified by the civil affairs bureau.

If applicants want to seek health care at other hospitals than the designated hospitals (i.e. portability of the entitlement), they must get a grant from the county civil affairs bureau and the county health bureau before treatment, otherwise, MFA will not assist them. However, in most cases, the designated hospitals for MFA are the same as the designated hospitals for NCMS, which include township health centers, and county-level hospitals. This may be for fund rationing and management convenience considerations.

### Step 6. The delivery of assistance

MFA benefits are provided in two ways. One way is direct cash assistance from the funds after utilization of hospital services. In Xiaochang, the only county where there was no NCMS in 2006, this is the only way to receive assistance. In the other three counties with NCMS on the other hand, MFA provides its benefits in two ways. First, it pays the NCMS yearly premium on behalf of the households meeting the household characteristics criteria (as outlined in Table 2) to participate in NCMS. Second, the eligible households can also receive direct cash assistance of MFA if the out-of pocket medical expenditure is still high after reimbursement by NCMS.

**Figure 1:**
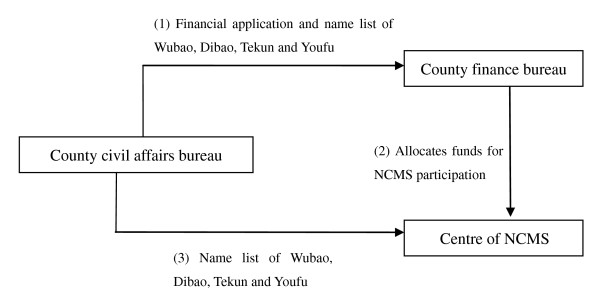
**Paying the NCMS premium for the applicants eligible for MFA**.

The basic mode of paying the NCMS premium for the eligible for MFA in Hongan, Fushun and Langzhong where NCMS was already implemented in 2006, is illustrated in Figure 1.

**Figure 2:**
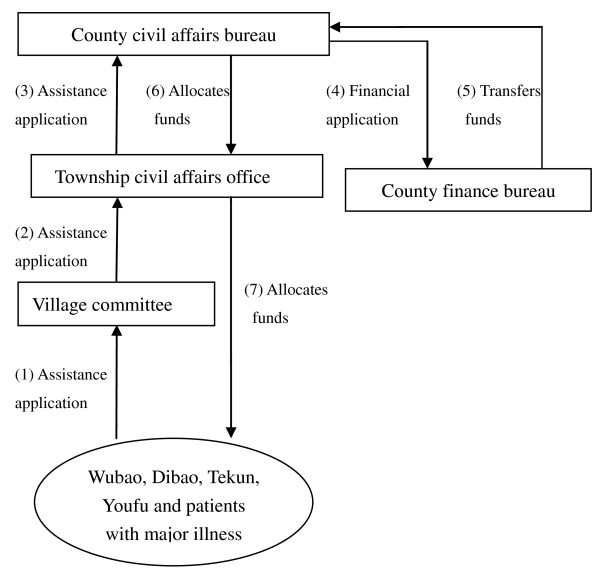
**Direct cash assistance in the four counties in 2006**.

The mode of direct cash assistance in the four counties is illustrated in Figure 2. Usually the whole process from applying for MFA till acquiring cash assistance takes several days to half a year. This mainly depends on the frequency of approval. Once approved, the beneficiaries can get the money within several days. The assistance standard and threshold of MFA are more rigorously specified in Xiaochang and Hongan than in Fushun and Langzhong.

Table 4 provides some more detail on the exact nature of the ex post financial assistance which can be received from MFA. The fact that MFA reimburses hospital services de facto sets other criteria for the assistance: households must have been able to afford hospital utilization. In addition, the generosity of the MFA has limits: there are thresholds, ceiling and the reimbursement is only partial. Households will therefore only get MFA assistance if they were able to pre-finance the part of the costs to be reimbursed by MFA and to fully cover the costs of the non-reimbursed part of the bill.

**Table 4:** The assistance standards & package of MFA in the four counties in 2006

	Xiaochang	Hongan	Fushun	Langzhong
Premium for NCMS (Yuan per capita)	Not relevant	15	10	10
Direct cash assistance rate (of out-of-pocket expenditure)	30%	20% ~30%	15%~30%	non-specific
Threshold (Yuan per capita)	1000	1000	non-specific	non-specific
Ceiling (Yuan per capita)	2, 000	3, 000	3, 000	3, 000

We have also mentioned that the list of allowed hospitals can be limited. In counties where the county affair bureau and county health bureau have designated hospitals, households must know which hospitals are covered. The regulation states that these hospitals are required to set up a special office for MFA under the supervision of the county health bureau, to make public the detailed regulations of assistance, and to provide high quality services to the patients covered by MFA. In Xiaochang and Hongan, the designated hospitals are allowed to offer some extra aid for patients enrolled in MFA. With the relevant certifications, they can be registered and diagnosed in the hospitals for free, just having to pay part of the fees for accommodation in hospital. Moreover, for fees of each item of nursing, surgery, examination and medicine, there is a 10%~20% exemption of the part exceeding 100 Yuan.

### Preliminary impact of MFA in the 4 counties

We try to assess the preliminary effect of MFA drawing on data from official documents and the RHS. We intend to assess whether the MFA targeting is appropriate and whether the assistance really benefits the beneficiaries. Do poor people really use MFA and is the assistance substantial enough to make a difference?

In China, the central government sets a 'National poverty line' to define 'poor households' in the whole country and the threshold has gone up in line with the country's price index. In 2006, the 'National poverty line' was set at an annual income of 683 Yuan per capita. Since the budget of MFA is very limited (we will show the exact figures in the paragraph below), the county civil affairs bureau cannot enroll all the poor households (as defined by this 'National poverty line') in MFA. Hence, as we mentioned in step 2, the county civil affairs bureau used the records of four other social assistance schemes (Wubao, Dibao, Tekun and Youfu) to define 'the MFA poor'. The four social assistance categories of households are identified by the county civil affairs bureau by assessing their economic status. The households included in these four categories amount to the most destitute among the poor households identified by the county government using the 'National poverty line'. The county civil affairs bureau enrolled all the households that appeared on the name lists of Wubao, Dibao, Tekun and Youfu in the MFA scheme. According to the official documents of the four counties, the coverage rate of MFA in 2006 ranged from 17.8% to 24.1%. The coverage rate in Langzhong county was higher than the rates in other counties (see table 5).

**Table 5:** The coverage rate of MFA in the four counties in 2006

County	Poor households identified by county government	Registered in MFA	
	
	***N(households)***^**a**^	***N(households)***^**b**^	*Percentage(%)*
Xiaochang	14336	2553	17.8
Hongan	27400	5320	19.4
Fushun	not available	4506	----
Langzhong	19355	4667	24.1

^a ^Figures of column 1 were collected by the county statistical bureaus according to the 'National poverty line'

^b ^Figures of column 2 were equal to the amount of the Wubao, Dibao, Tekun and Youfu households.

In table 6 we provide some fragmentary data on MFA assistance to the rural poor in the four counties, as of 2006.

**Table 6:** The utilization of MFA in the four counties in 2006

	Xiaochang	Hongan	Fushun	Langzhong
Budget (Yuan)	810, 000	490, 000	1, 243, 000	1, 646, 000
Budget/Rural population (Yuan)	1.45	0.90	1.50	2.74
Budget/main eligible population (Yuan)	Not available	32.67	95.38	149.09
Contribution of MFA for NCMS premium (Yuan)	Not relevant	236, 745	131, 810	140, 000
Persons enrolled in NCMS due to MFA	Not relevant	15, 783	13, 181	11, 218
Beneficiaries of direct cash assistance (person-time)	263	179	338	688
Average benefit/beneficiary (Yuan)	1, 883	2, 200	700	1, 293
Beneficiaries per year/Number of households officially enrolled in MFA	0.10	0.03	0.07	0.15

The MFA budget of Xiaochang and Hongan (Hubei province) is lower than the MFA budget of Fushun and Langzhong (Sichuan province), but the benefit amount per actual beneficiary in Xiaochang and Hongan is higher than in Fushun and Langzhong. The number of people enrolled in NCMS with help of MFA is relatively similar in Hongan, Fushun and Langzhong, but the total expenditure for paying the NCMS participation fee is substantially higher in Hongan than in Fushun and Langzhong due to the higher NCMS participation fee per person in Hongan. In Hongan, a substantial share of the MFA budget is in fact allocated to the NCMS premium.

It is interesting to compare this information from the routine information system with the data collected from households. According to RHS, the coverage rate of the households is extremely low (6.9%). The difference between the respective coverage rates in table 5 and table 7 (self-reported welfare status) could be due to the limited awareness of the MFA scheme among rural poor households --beneficiaries who were provided the NCMS premium through MFA funds were perhaps not aware of the link with MFA. In practice, the county civil affairs bureau directly allocated MFA funds to the payment of the NCMS participation fee if a household's name appeared on the name list of Wubao, Dibao, Tekun and Youfu households. The bureau did not bother to inform the beneficiaries.

**Table 7:** The poor households reporting a welfare status in RHS (2006)

County	Poor households	Registered in MFA	
	
	*N(households)*	*N(households)*	*Percentage(%)*
Xiaochang	202	9	4.5
Hongan	163	10	6.1
Fushun	129	11	8.5
Langzhong	57	8	14.0
Total	552	38	6.9

In the RHS data, only 20 households reported to have received direct cash assistance from the MFA, which accounted for more than half of the 38 households covered by MFA in the sample. So the rate of MFA utilization among households aware of their MFA status is rather high; this is encouraging. Among the 20 households, the minimum benefit was 50 Yuan, the maximum benefit was 3000 Yuan and the median was 1100 Yuan. Among the 18 households who did not get assistance, 15 households had members who got major illness and also incurred medical expenditure. Given that the RHS was conducted in early 2007 and the application for assistance did take some time, these household may have been in the process of application at the time.

Our interviews with local officials offered another source of information on the preliminary effect and performance of MFA. Officials in the four counties all claimed that MFA is a really good scheme, as it evidences government concern for the poor with major illness and is helpful in relieving poverty due to major illness. However, they admitted that MFA could not fully solve the poverty trap caused by major illness, due to the limited funds available. For example, medical expenditure for some major illnesses such as leukemia and uremia was huge, but the financial relief provided by MFA was often woefully inadequate. When the poor are unable to prepay for hospitalization, MFA is incapable of paying either. Besides, for those who need pre-assistance, the MFA scheme is ineffective due to the post-assistance mode, which only provides assistance after the poor person has paid her/his bills in the hospital. In the officials' view, a better MFA scheme thus mainly depends on better MFA policy making and a higher budget.

## Discussion

Progress towards universal coverage will require specific schemes to help the poorest to avail the services they need. The purpose of this paper was mainly to review the design and implementation of MFA in four counties in Hubei and Sichuan province, using the analytical framework developed by Meessen and Criel [15]. The paper also aims to help governments at different levels to fine-tune the implementation of MFA. From the six steps, discerned by the framework, arises the following picture for MFA in the counties.

**Step 1: **While formulated at national level, the MFA policy allows decentralized governments quite some leeway to operationalize the strategy. Nevertheless, one can observe great homogeneity in the four countries in terms of implementation. This is particularly striking for the roles of the different bureaus and the modesty of the budgets allocated to the policy. This limited financial commitment is an obvious first reason why MFA does not reach all the poor rural households struggling with their medical bill. It can also be interpreted as an indication that local governments do not consider medical assistance to the poorest households a top priority.

**Step 2: **We observe also great homogeneity in the four counties in terms of eligibility criteria: they are invariably very restrictive. Poverty hinges on an administrative definition, i.e. being a Wubao, Dibao, Tekun or Youfu household. The thresholds of these social assistance schemes are very low, hence, many poor households are excluded from MFA. In other words, a lot of the proclaimed targeted benefiaries of the MFA policy are already lost at the eligibility definition step. We have seen that MFA decentralized operators were aware of the rather restrictive character of these eligibility criteria. They often tried to use the medical expenditure criteria to be more inclusive. However, this flexibility could also be used to ration access to the MFA.

The main stated purpose of MFA is to help rural poor people cope with major illness. We have seen that MFA endorses a view that major illness is a problem handled at least at hospital level, with a bias towards inpatient care. This is another reason why the MFA may miss its target. There has been some debate lately in the Chinese literature on the appropriate definition of major illness [19-21]. Lu stated that the definition of major illness should be commensurate with the amount of medical expenditure which a household cannot afford; in other words, to the extent that it renders the household poor [22]. So the definition of 'major illness' for MFA needs to be broadened. All health events with a possible major impact on household livelihood should be included. This shows in fact the relative character of the major illness concept: major relative to what? To reach its objectives, the MFA should (1) cover high medical bills, either for acute illness, chronic care or even diagnosis; (2) be capable to adapt, to some extent, its eligibility threshold and assistance according to the socio-economic status of the applicant (for the very poor, basic outpatient care can already be inaccessible). Moreover, the economic burden of illness should be measured at the household level, not just at the individual level. A solution could be that MFA intervenes more generously above a certain threshold for the total health care expenditure of the households for a given period.

**Step 3: **Official documents we reviewed suggested that the county governments had made some effort to inform the stakeholders, especially the poor that were targeted by MFA. However, RHS has revealed that only a small fraction of the poor households were actually aware of MFA. The semi-structured interviews we conducted allowed us to explain at least to some extent this puzzling observation. Some officials said that the local governments were reluctant to advertise MFA due to limited funds. They were afraid that the budget of MFA would not suffice if too many poor people applied for assistance. Hence, many poor people did not know that MFA was intended to help them cope with major illness. Publicizing MFA or not was really a dilemma for local governments. Informing stakeholders is a vital step where many poor households can end up being excluded due to a lack of information: those who have no access to the media and other sources of information will not hear about the program; it is then impossible for them to take the required subsequent steps, e.g. submit an application.

**Step 4: **Identification processes in the four counties were fairly similar. MFA is an ex post assistance scheme: the identification is based on certificates and documents proving that the household deserves to be partly reimbursed for its expenditure. We have seen that the village cadres play a key role in the identification: they make the household assessment of poverty and diseases and have a discussion to identify the poor households. We have not collected evidence on possible favoritism, an issue sometimes reported with community-based targeting [23].

**Step 5: **We have seen that the decision by the village civil affairs cadre or the village cadre does not guarantee that the program will actually assist the household. Due to the extremely limited funds, MFA managers ration the assistance. In Sichuan, for instance, poor households which received NCMS reimbursement get precedence for MFA assistance over other poor households. Households claiming assistance are also ranked. Other citizens can also contest the eligibility of someone (lists of people requesting reimbursement are made public). The requirement to provide many documents, the uncertainty on the final assistance and the obligation to rapidly claim the reimbursement are certainly possible causes for losing some of the target group. Having said this, a strength of MFA is that it could become a rights-based policy. We have seen that at least for Wubao and Tekun households, MFA has become an extension of their welfare status.

**Step 6: **We have seen that MFA could provide two types of benefits: a premium for enrolment in NCMS and reimbursement of medical bills. The first option has some leverage effect: being a member of the NCMS scheme does indeed allow the household to get more reimbursement for their hospital expenditure. Yet, this will occur only if the household uses the inpatient services. The problem, identified also by our key informants in Sichuan, is that MFA does not remove the barriers to hospital utilization at all. The scheme focuses on reducing ex post the financial burden of hospital utilization: it is still the household itself that has to mobilize resources to reach the hospital, e.g. transport, cash to pay medical bills. By design, it is more a scheme trying to protect consumption than to enhance good health. The second objective would indeed require providing an entitlement guaranteeing assistance ex ante and ex post, substantial enough for convincing households to go to the hospital. If no assistance is provided ex ante, paying NCMS premiums through MFA could in fact be regressive, as the pooled resources will benefit only those able to afford hospital utilization. As correctly identified by officials in Sichuan counties, the MFA loses many poor because of its ex post mode of assistance. This situation amounts to so-called 'negative cross-subsidization'. From a systems perspective, community health insurance may result in poorer groups contributing to their health care costs to a greater extent than richer groups who are able to access public services, and thus may be inequitable with respect to payment [24]. Jacobs et al. reported that the outpatient consultation and hospitalization rates were much more lower in the Health equity funds (HEF) member population than that in Community-based health insurance (CBHI) member population, which implied that there was an obvious risk of cross-subsidy from the HEF to the CBHI fund when HEF offered the same premium for its members to the CBHI fund pool [25]. Annear et al. also explored the equity and effectiveness in purchasing health insurance premiums for the poor who were enrolled in Health equity funds (HEF) in Cambodia and Laos, and they concluded that it was unlikely that horizontal equity (equal access for the same health need) will be achieved while service utilization remains lower amongst HEF than voluntary CBHI members, i.e. government and donor subsidies intended for the poor may in fact be captured in part by the non-poor [26].

With respect to the preliminary impact of MFA in these counties, the general picture was assistance to a disappointingly low number of poor patients, although we observed some variation across the four counties. According to the semi-structured interviews, the main reason for this phenomenon was limited funding. China is a quite decentralized system in terms of government funding. Most of the responsibility for financing MFA rests upon local governments, particularly the counties. This has contributed greatly to the scarcity or even absence of social protection programs in rural areas [12].

The results show that at the time of research MFA did not reach its objectives. The main cause was the scarcity of resources dedicated to the program. Different mechanisms were and probably still are in place to ration the assistance. In the end, too few households benefited from the assistance and the assistance was probably too meager to make a real difference.

This paper provided also an opportunity to assess - in rural China - the validity of the framework developed by Meessen & Criel for the health equity fund experience in Cambodia. This question has relevance beyond the Chinese context: the question of how to assist the poorest in their utilization of health services is indeed a universal one. In a previous paper, Meessen et al. had also partly used this framework to compare the user fees exemption policy in Uganda with health equity funds in Cambodia. Although the two policies took place in very different countries and displayed many differences in implementation, the basic idea or rationale of this framework proved useful to analyze health assistance schemes [27]. However, to some extent, the framework has reached its limit in terms of description of the process (descriptive power). The underlying sequence in the framework may not apply to all social assistance schemes. For instance, with a scheme like MFA, the entitlement comes after the utilization of the health service. The framework seems however helpful to understand where a targeting program loses its target group (analytical power) and how one can improve the performance of the program (prescriptive power).

### Ways forward

China is a country experiencing very rapid economic development and policies are adapted continuously. In June 2009, the Ministry of Civil Affairs issued the 'Suggestion on further improving Medical Financial Assistance' [28] to improve the MFA scheme in terms of stable sources of funding, standard operation of management, clear effect of assistance and convenience of application. To achieve these objectives, some important measures were recommended to local governments. Firstly, MFA should be funded by all kinds of resources. The local government, especially the provincial level government should take responsibility for increasing funds. The central government will offer special funds as a subsidy to poor regions, for instance, to some poor counties in western China. The local governments should also encourage social organizations and individual persons to fund MFA through charity or social contributions. Secondly, eligibility should be defined reasonably. In addition to covering Dibao and Wubao households, MFA should also consider poor households that have members with major illness and high medical expenditures. Thirdly, the poor should be assisted in different ways. Although MFA should mainly cover inpatient expenditures of beneficiaries, it should also assist outpatients who need to take medicine for a long time due to chronic disease and beneficiaries who get emergency treatment. Fourthly, assistance proposals need to be formulated reasonably. Local governments are required to formulate assistance proposals according to the amount of MFA funds, decrease or remove the deductible, increase the ceiling and the actual assistance proportion. In addition, some other changes were mentioned in the paper, such as simplifying the procedure of application, strengthening the supervision of funds and keeping in check an unreasonable increase of medical expenditure. In July 2009, the Ministry of Health, Ministry of Civil Affairs and Ministry of Finance jointly issued the 'Suggestion on consolidating and improving the NCMS' [29] which emphasized linkages between NCMS and MFA in terms of policy design, management and expenses. The most remarkable term in this paper was the term 'One-stop Service', implying a united service platform for MFA and NCMS. In order to improve the convenience of assistance by MFA and NCMS for the beneficiaries who are covered by both MFA and NCMS, the central government encourages the local government to create a combined database system of the two schemes if possible. Through this database system, the poor will be able to get immediate assistance and reimbursement when they settle the account at health care facilities.

In August 2009, we visited Langzhong again to follow up the implementation of MFA. According to the interview with managers of this scheme, some problems described in this paper had been addressed in Langzhong, but some key issues still existed, such as the very limited funds, the low coverage rate and no united service platform of MFA and NCMS.

The real future of the MFA scheme lies probably in comprehensively addressing all the barriers the poor encounter when trying to access a basic package of curative services. Time will tell whether social health assistance really takes off in rural China.

## Competing interests

The authors declare that they have no competing interests.

## Authors' contributions

XM, JZ, BM, YY and XR contributed to the design of this study. JZ, MB, KD and XT performed data analysis. All authors contributed to the draft writing of the manuscript. MB and KD also contributed to polishing this manuscript. All authors have read and approved the final manuscript.
